# Genetic evidence for causal link between systemic inflammation and dental caries risk: A bidirectional Mendelian randomization study

**DOI:** 10.1097/MD.0000000000050025

**Published:** 2026-07-31

**Authors:** Zhe Xu, Xiaofen Liu, Fengzhen Lei, Ziyang Hu, Ping Shi

**Affiliations:** a Department of Stomatology, Shenzhen Longhua District Central Hospital, Shenzhen,China.

**Keywords:** cytokines, Genome-wide Association Study, oral health, SNPs

## Abstract

The causal relationship between systemic inflammation and dental caries remains unclear. This study aimed to investigate the bidirectional causal association between circulating cytokines and the risk of dental caries. A bidirectional 2-sample Mendelian randomization (MR) study was conducted. Genetic instruments for 132 circulating cytokines were obtained from 2 large-scale genome-wide association study consortia (n = 8293 and n = 14,824). Summary statistics for dental caries were sourced from the FinnGen consortium (n = 1,95,395). The inverse variance weighted method was used for the primary analysis, supplemented by a range of sensitivity analyses (e.g., MR-Egger, weighted median, MR-PRESSO) to assess the robustness of the findings and detect horizontal pleiotropy. Genetically predicted higher levels of several cytokines were causally associated with an increased risk of dental caries, including CXCL9 (odds ratio [OR] = 1.25, 95% confidence interval [CI]: 1.05–1.49 and CTACK (OR = 1.15, 95% CI: 1.04–1.27). Conversely, higher levels of IFN-γ (OR = 0.79, 95% CI: 0.65–0.96), IL-17 (OR = 0.83, 95% CI: 0.71–0.97), and RANTES (OR = 0.85, 95% CI: 0.76–0.96) were associated with a reduced risk. In the reverse analysis, genetic liability for dental caries was causally linked to increased levels of Artemin and decreased levels of Cystatin D and IL-2RA. Sensitivity analyses confirmed the robustness of these associations. Our findings reveal a bidirectional causal relationship between specific inflammatory cytokines and dental caries. This suggests that systemic inflammatory pathways both contribute to and are influenced by the pathogenesis of dental caries, highlighting potential biomarkers and therapeutic targets.

## 1. Introduction

Dental caries is the most prevalent noncommunicable disease and a major global public health challenge.^[1]^ Oral diseases affect an estimated 3.5 billion people, with untreated caries in permanent teeth being the single most reported health condition worldwide.^[2,3]^ While established risk factors are well-recognized, the etiological landscape is incomplete, making it imperative to explore novel biological pathways to inform more effective preventive strategies.

Systemic inflammation, mediated by circulating cytokines, has emerged as a biologically plausible but unconfirmed contributor to caries pathogenesis.^[4]^ Mechanistic studies demonstrate that the host immune response to cariogenic bacteria within the dental pulp involves the localized production of pro-inflammatory cytokines, including interleukin-1β (IL − 1β), interleukin-6 (IL − 6), and tumor necrosis factor-α (TNF-α), which drive inflammation and subsequent tissue degradation.^[5]^ This biological rationale is supported by observational data, where studies have reported positive associations between salivary concentrations of cytokines such as IL-6 and IL-8 and the presence of cavitated carious lesions in children.^[6]^ These findings suggest that an individual’s inflammatory profile may influence their susceptibility to or progression of dental caries.

However, whether the observed association between inflammatory cytokines and dental caries is causal remains uncertain due to the inherent limitations of conventional observational study designs.^[7]^ Such associations are susceptible to bias from unmeasured or residual confounding, where factors like diet, oral hygiene practices, and socioeconomic status influence both systemic inflammation and caries risk independently. More critically, the relationship is prone to reverse causation; the infectious and destructive nature of the carious process is itself a potent inflammatory stimulus, meaning elevated cytokine levels may be a consequence of the disease rather than a cause.^[8]^ These limitations preclude the establishment of causality from existing evidence, creating a critical knowledge gap. Advanced clinical treatments, such as laser surgery, can address the local inflammatory outcomes of oral diseases.^[9,10]^ However, these approaches are directed at the consequences, leaving the causal contribution of systemic inflammatory predispositions to be determined.

Mendelian randomization (MR) is a genetic approach that uses genetic variants as IVs to infer causality, thereby mitigating confounding and reverse causation.^[7,11]^ Its validity rests on 3 core assumptions. While a prior MR study provided preliminary evidence for the effect of cytokines on a composite “oral disease” outcome,^[12-14]^ the specific causal link with dental caries and the potential for reverse causality have not been investigated. Therefore, our study utilizes a bidirectional MR framework to systematically dissect the 2-way causal relationship between systemic inflammation and dental caries.

Therefore, this study employed bidirectional MR to systematically assess the 2-way causal relationship between 132 circulating inflammatory cytokines and the risk of dental caries. This research aims to provide high-quality genetic evidence to inform future mechanistic studies and the development of novel strategies for preventing and managing this disease.

## 2. Materials and methods

### 2.1. Study design

**Figure 1.**
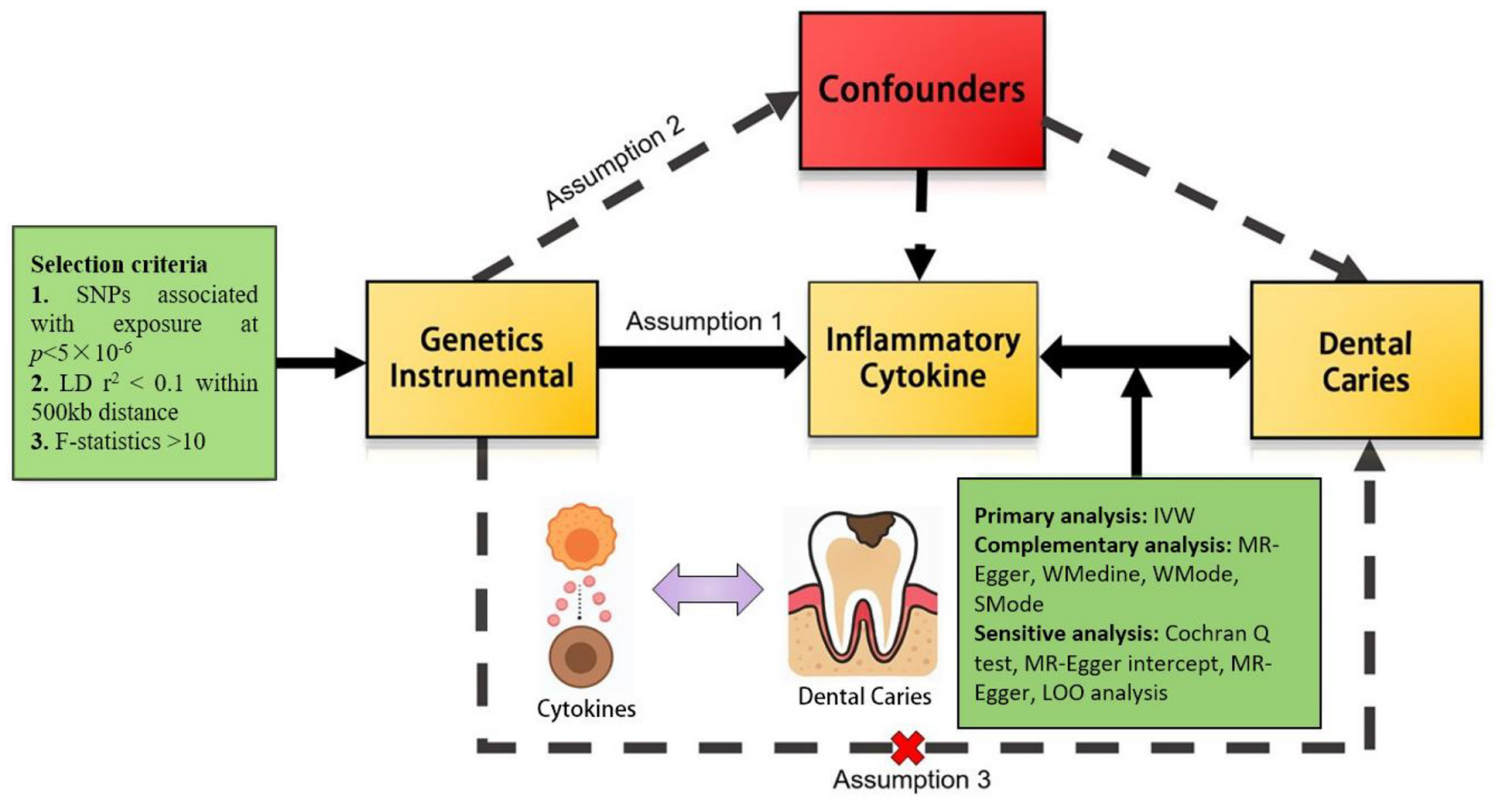
Diagram of the bidirectional MR analysis. Assumption 1, genetic instruments are strongly associated with the exposures (inflammatory of interest); Assumption 2, genetic instruments are independent of confounding factors; Assumption 3, genetic instruments are not associated with the outcome and affect the outcome (dental caries) only via exposures. Two cohorts of cytokines were included in this study. IVW = inverse variance weighted, LD = linkage disequilibrium, LOO analysis = leave-one-out analysis, MR-PRESSO = Mendelian Randomization-Pleiotropy RESidual sum and outlier, SMode = simple mode, SNP = single nucleotide polymorphisms, WMed = weighted median, WM = weighted mode.

This bidirectional 2-sample MR study was designed to investigate the causal relationship between circulating cytokines and dental caries using single nucleotide polymorphisms (SNPs) as instrumental variables (IVs). The overall study framework is outlined in Figure 1. The validity of any MR analysis relies on 3 core assumptions: the IVs must be strongly associated with the exposure; the IVs must be independent of any confounders of the exposure-outcome relationship; and the IVs must affect the outcome exclusively through their effect on the exposure.^[15]^

The first assumption was addressed during the IV selection process. The remaining 2 assumptions, which cannot be directly verified, were assessed through a suite of sensitivity analyses designed to detect and account for horizontal pleiotropy (Fig. 1). This study was conducted and reported in accordance with the Strengthening the Reporting of Observational Studies in Epidemiology using Mendelian Randomization (STROBE-MR) guidelines. This study was approved by the Ethics Committee of our Hospital (2023-011-02).

### 2.2. Cytokines data source

To analyze the up-to-date causal relationship between cytokines and dental caries, there are 2 cohort of cytokines included in this study (Fig. 1).

Cohort 1: The first dataset was a comprehensive genome-wide association study (GWAS) meta-analysis of 41 circulating cytokines in 8293 participants of European ancestry. This dataset combined genomic and proteomic information from the FINRISK 1997 study (n = 4608) and the FINRISK 2002 study (n = 1705), with additional data from the YFS study. Cytokine levels were quantified from EDTA-treated plasma (FINRISK 1997), heparinized plasma (FINRISK 2002), and serum (YFS) using the Bio-Plex 200 system. The genetic association estimates were adjusted for age, sex, BMI, and principal components of genetic ancestry to mitigate confounding. A summary of the cytokine GWAS data is provided in Table S1, Supplemental Digital Content 1.

Cohort 2: The second dataset was sourced from the SCALLOP Consortium, providing GWAS summary statistics for 91 circulating inflammatory proteins measured with the Olink Target-96 Inflammation panel (Table S2, Supplemental Digital Content 2).^[16]^ Genotyping was performed on SNP arrays, and data were subsequently imputed using the 1000 Genomes or HRC reference panels. This analysis aggregated data from 14,824 individuals to map protein quantitative trait loci (pQTLs) at a significance threshold of *P* ≤ 5 × 10^−10^.^[16]^ This study’s findings were juxtaposed with those of the ARISTOTLE study, with clear definitions of pQTLs that reflect associations with protein abundance.^[17,18]^ Protein variance was determined using a standardized formula, and conditional analyses were conducted with GCTA to identify independent signals.^[16]^

### 2.3. Dental caries data source

Genetic summary statistics for dental caries were obtained from the FinnGen consortium. FinnGen is a large-scale Finnish biobank study that links genomic data from 1,95,395 participants with comprehensive health data from national registers. Disease endpoints were defined using Finnish versions of international classification systems. Genotyping was conducted on Illumina and Affymetrix arrays, followed by rigorous quality control and imputation to the latest human reference genome.

### 2.4. Instrument selection

Genetic instruments for each cytokine were selected based on their independent and significant association with the corresponding protein level. Initially, we applied a strict genome-wide significance threshold (*P* < 5 × 10^−8^). However, after clumping, this yielded fewer than 3 eligible SNPs for most cytokines in the first cohort, which is insufficient for robust pleiotropy assessment. Consequently, we adopted a more liberal significance threshold of *P* < 5 × 10^−^⁶, a common practice in MR studies.^[15]^

To ensure instrument independence, selected SNPs were clumped based on linkage disequilibrium, retaining the SNP with the lowest *P*-value while removing those in linkage disequilibrium (*r*^2^ > 0.1 within a 500 kb window). To mitigate potential weak instrument bias, we calculated the *R*^2^ and *F*-statistic for each SNP using established formulas that incorporate the effect size (β), standard error (se(β)), effect allele frequency, and sample size (N). Any SNP with an *F*-statistic below 10 was excluded. Finally, the instrument SNPs were harmonized with the outcome data, and any palindromic SNPs or those with ambiguous allele alignments were removed to ensure a robust analysis.^[19]^


$$R^{2}=\frac{2\times \beta^{2}\times EAF\times \left(1-EAF\right)}{\left[2\times \beta^{2}\times EAF\times \left(1-EAF\right)+2\times (se{\left(\beta \right)}^{2}\times N\times EAF\times \left(1-EAF\right)\right]}$$

$$\begin{array}{c} F=\frac{N-k-1}{k}\times \frac{R^{2}}{1-R^{2}} \end{array}$$

### 2.5. Mendelian randomization analysis

We conducted a bidirectional MR analysis to evaluate the causal effects in both directions: from cytokines to dental caries and from dental caries to cytokines. This was performed for both the 41-cytokine cohort and the 91-cytokine cohort.

The primary causal estimates were derived using the inverse variance weighted (IVW) method, which combines the Wald ratio for each SNP into a meta-analytic summary. In the absence of horizontal pleiotropy, the IVW estimate is unbiased.^[20]^ We then employed several sensitivity analyses to assess the robustness of our findings. MR-Egger regression was used to detect and adjust for directional pleiotropy via its intercept term.^[12]^ The weighted median method provides a consistent causal estimate even if up to 50% of the instruments are invalid.^[21]^ The weighted mode method offers an alternative estimate that is robust even when the InSIDE (Instrument Strength Independent of Direct Effect) assumption is violated.^[22]^ Although the simple mode method may lack precision, it generally exhibits reduced bias compared to other methods.^[22]^ Finally, we used the MR-PRESSO (Pleiotropy RESidual Sum and Outlier) test to identify and correct for pleiotropy by removing outlier SNPs.^[23]^

### 2.6. Statistical analysis

All statistical analyses were performed in R (version 4.3.3; R Foundation for Statistical Computing, https://www.R-project.org/) using the “TwoSampleMR” and “MRPRESSO” packages.^[24]^ Heterogeneity among the IVs was quantified using the modified Cochran’s *Q* statistic. Horizontal pleiotropy was assessed using the MR-Egger intercept and the MR-PRESSO global test.^[25]^ To confirm the assumed causal direction (i.e., that the IVs explain more variance in the exposure than in the outcome), we applied the Steiger test.^[26]^ For the binary outcome of dental caries, causal effects were calculated as odds ratios (ORs) with 95% confidence intervals (CIs) per standard deviation increase in the level of each biomarker.

## 3. Result

### 3.1. Causal effect of inflammatory cytokines on the risk of caries

After excluding unmatched SNPs and finding proxies in outcome data, we performed an MR analysis. The *F*-statistics for the IVs were all >10, indicating no potential bias from weak instruments (Table S3, Supplemental Digital Content 3). The statistical results for the MR analysis are shown in Table S4, Supplemental Digital Content 4.

For the main MR analysis using the IVW method, we identified several cytokines with a potential causal effect on dental caries. Risk factors included C-X-C motif chemokine 9 (CXCL9; CXCL9 → caries: OR = 1.251, 95% CI: 1.051–1.489, *P* = .012), Natural killer cell receptor 2B4 (CD244; CD244 → caries: OR = 1.196, 95% CI: 1.026–1.394, *P* = .022), Cutaneous T-cell attracting chemokine (CTACK; CTACK → caries: OR = 1.150, 95% CI: 1.039–1.272, *P* = .0068), and C–C motif chemokine 25 (CCL25; CCL25 → caries: OR = 1.136, 95% CI: 1.010–1.277, *P* = .033).

**Figure 2.**
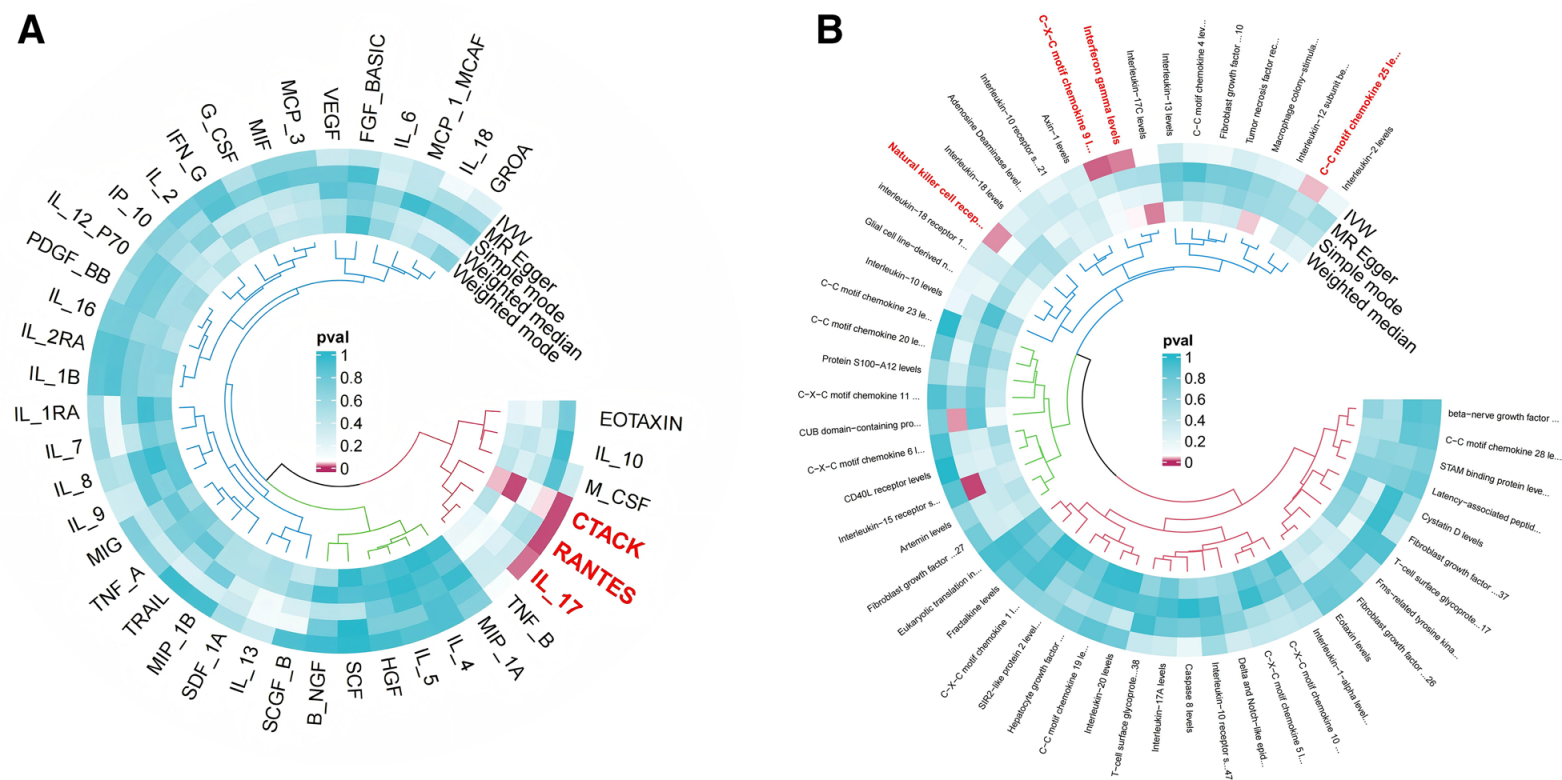
Preliminary MR analyses for the associations between cytokines and the risk of dental caries. (A) MR analysis results for 41 circulating cytokines in Cohort 1. (B) MR analysis results for 91 circulating inflammatory proteins in Cohort 2. The circles from the outer to the inner represent the IVW, MR-Egger, simple mode, weighted median, and weighted mode, respectively. The shades of color reflect the magnitude of the *P*-value as labeled inside the circles. IVW = inverse variance weighted, MR-Egger = MR-Egger regression.

Conversely, some cytokines were identified as protective factors. These included regulated on activation, normal T cell expressed and secreted (RANTES; RANTES → caries: OR = 0.853, 95% CI: 0.761–0.957, *P* = .0065), interleukin-17 (IL-17; IL-17 → caries: OR = 0.830, 95% CI: 0.714–0.965, *P* = .015), and interferon-gamma (IFN-γ; IFN-γ → caries: OR = 0.787, 95% CI: 0.645–0.961, *P* = .018; Fig. 2 and Table 1).

**Table 1:** IVW result of cytokines on dental caries in 2 cohorts.

Cohort	Exposure	NSNP	B	SE	*P*-value	OR (95% CI)
1	IL-17	10	–0.186	0.077	.015	0.830 (0.715–0.965)
1	CTACK	13	0.139	0.051	.0068	1.150 (1.039–1.272)
1	RANTES	10	–0.159	0.058	.0065	0.853 (0.761–0.957)
2	CXCL9	19	0.224	0.089	.0116	1.251 (1.051–1.489)
2	CD244	21	0.179	0.078	.0224	1.196 (1.026–1.394)
2	IFN-γ	10	–0.239	0.101	.0184	0.787 (0.645–0.961)
2	CCL25	27	0.127	0.060	.0329	1.136 (1.010–1.277)

CCL25 = C–C motif chemokine 25, CD244 = natural killer cell receptor 2B4, CI = confidence interval, CTACK = cutaneous T-cell attracting chemokine, CXCL9 = C-X-C motif chemokine 9, IL-17 = interleukin-17, NSNP = number of single nucleotide polymorphisms, OR = odds ratio, RANTES = regulated on activation, normal T cell expressed and secreted, SE = standard deviation.

**Figure 3.**
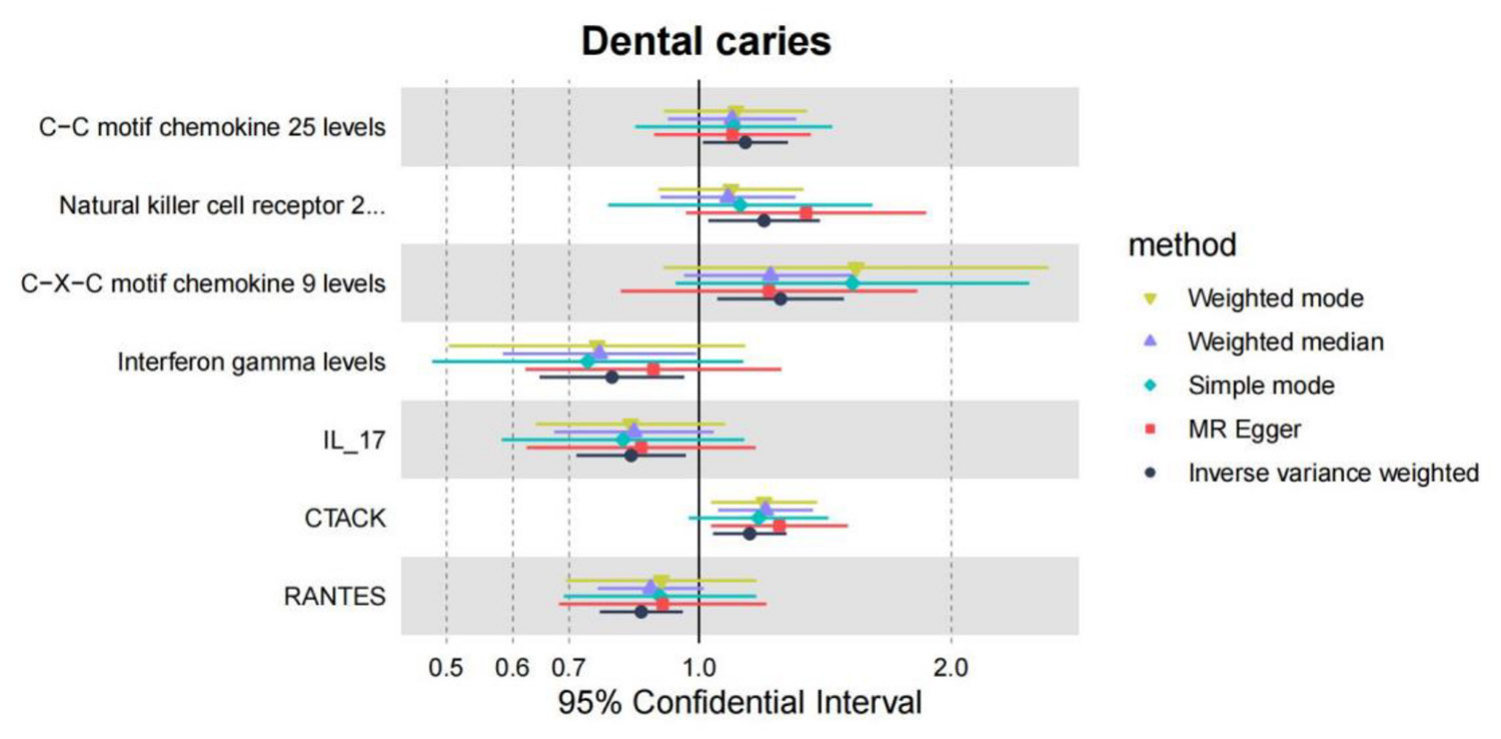
Forest plot of MR analysis. Forest plot to visualize the causal effect of cytokines on the risk of dental caries risk factors by inverse variance weighted, MR-Egger regression, weighted median, weighted mode method, and simple mode. CXCL9, CD244, CTACK, and CCL25 may increase the risk of dental caries. RANTES, IL-17 and IFN-γ may decrease the risk of oral cancer. CCL25 = C–C motif chemokine 25, CD244 = natural killer cell receptor 2B4, CTACK = cutaneous T-cell attracting chemokine, CXCL9 = C-X-C motif chemokine 9, RANTES = regulated on activation, normal T cell expressed and secreted, MR-Egger = MR-Egger regression

**Figure 4.**
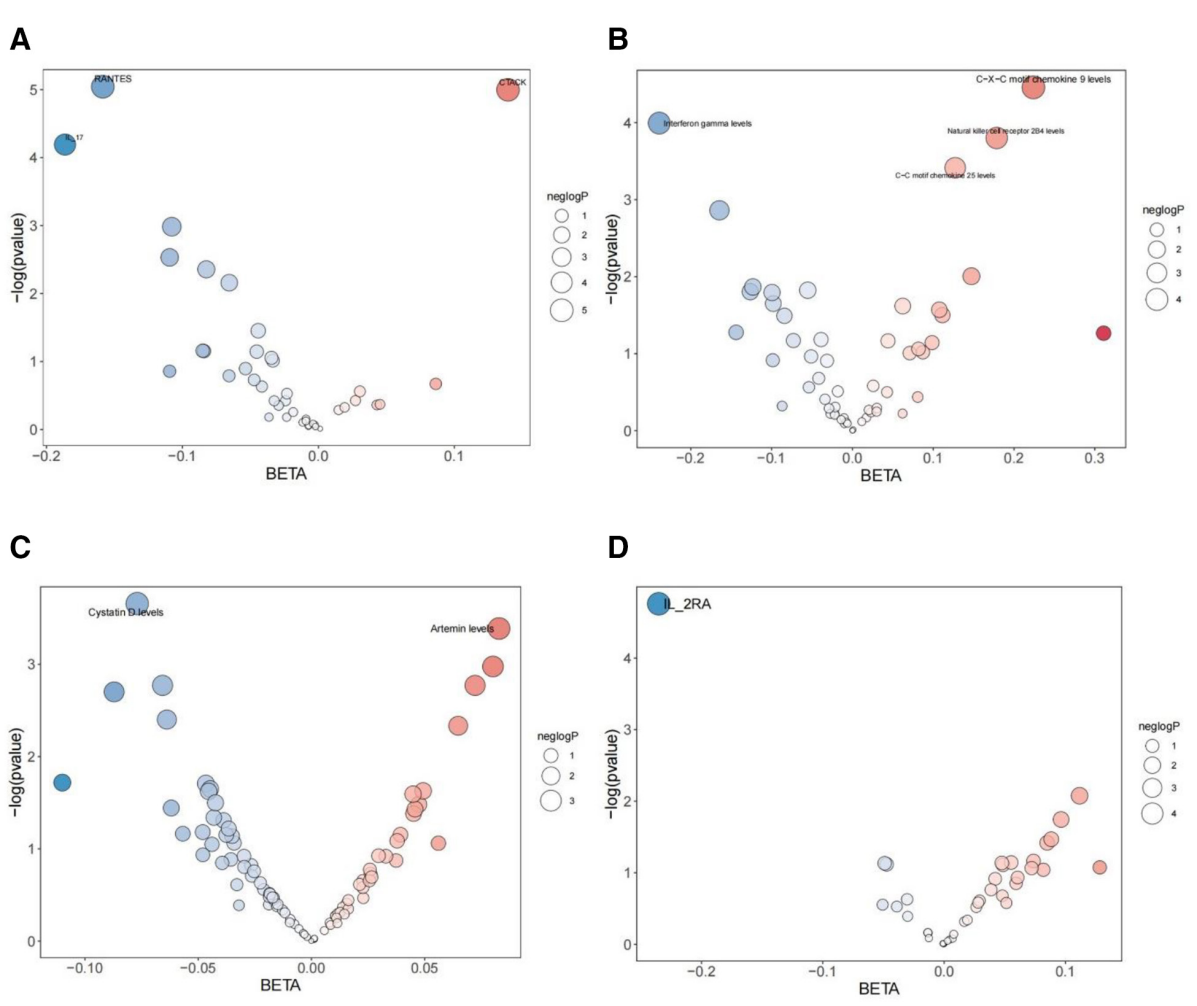
Bubble plots of MR analysis. A is a bubble plot of cohort 1 of MR analysis. B is a bubble plot of cohort 2 of MR analysis. C is a bubble plot of cohort 1 of reverse MR analysis. D is a bubble plot of cohort 2 of reverse MR analysis.

The analysis found no evidence of heterogeneity among the SNPs for these significant cytokines, as indicated by Cochran’s *Q* test. Furthermore, both the MR-Egger intercept and MR-PRESSO tests showed no significant pleiotropy, reinforcing the validity of our IVs (Table 2). The directionality was evaluated using the Steiger test and is shown in Table S5, Supplemental Digital Content 5. The forest plot from 5 different MR methods is shown in Figure 3. A leave-one-out analysis affirmed that no individual SNP introduced bias into the MR estimation (Fig. S1, Supplemental Digital Content 6). The funnel plots are shown in Figure S2, Supplemental Digital Content 7. Scatter plots for the identified cytokines across various tests are displayed in Figure S3, Supplemental Digital Content 8. Bubble plots of MR analysis are shown in Figure 4A, B. This multi-faceted approach ensured a thorough examination of the potential causal relationships.

**Table 2:** Sensitivity analysis of cytokines on dental caries.

Cohort	Exposure	IVW Cochran	MR-PRESSO	Egger intercept
1	IL-17	0.451	0.473	0.847
1	CTACK	0.225	0.293	0.336
1	RANTES	0.815	0.823	0.670
2	CXCL9	0.211	0.280	0.868
2	CD244	0.061	0.063	0.446
2	IFN-γ	0.606	0.660	0.464
2	CCL25	0.744	0.853	0.704

CCL25 = C–C motif chemokine 25, CD244 = natural killer cell receptor 2B4, CTACK = cutaneous T-cell attracting chemokine, CXCL9 = C-X-C motif chemokine 9, IFN-γ = interferon-gamma, IL-17 = interleukin-17, IVW = inverse-variance weighted, MR-PRESSO = Pleiotropy RESidual Sum and Outlier, RANTES = regulated on activation, normal T cell expressed and secreted.

### 3.2. Causal effect of risk of caries on inflammatory cytokines

In the reverse analysis, we investigated the causal effect of genetic liability to caries on cytokine levels. No evidence of weak instruments was identified, with all *F*-statistics > 10 (Table S6, Supplemental Digital Content 9). Analytical results from 5 methods are shown in Table S7, Supplemental Digital Content 10.

**Figure 5.**
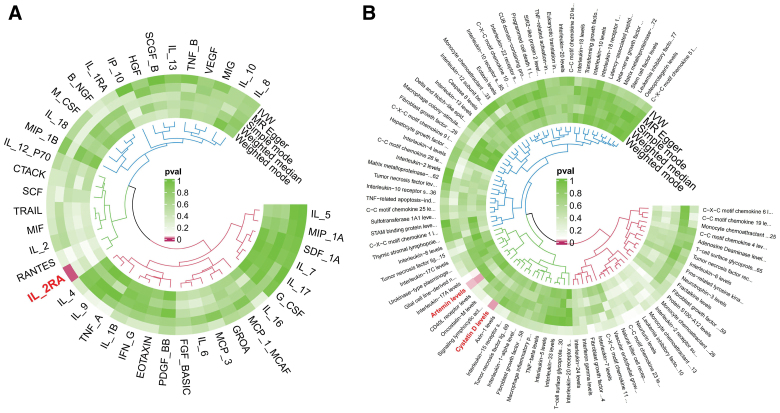
Preliminary MR reverse analyses for the associations between cytokines and the risk of dental caries. The circles from the outer to the inner represent the IVW, MR-Egger, simple mode, weighted median, and weighted mode, respectively. The shades of color reflect the magnitude of the *P*-value as labeled inside the circles. IVW = inverse variance weighted, MR-Egger = MR-Egger regression.

The results suggest that caries has a causal effect on the circulating levels of several cytokines. Specifically, caries was associated with an increase in Artemin (Caries → Artemin: OR = 1.086, 95% CI: 1.006–1.173, *P* = .034) and a decrease in Cystatin D (Caries → Cystatin D: OR = 0.926, 95% CI: 0.865–0.991, *P* = .026) and interleukin-2 receptor alpha (IL-2RA; Caries → IL-2RA: OR = 0.790, 95% CI: 0.663–0.942, *P* = .0086; Fig. 5 and Table 3).

**Table 3:** IVW result of dental caries on cytokines.

Cohort	Outcome	NSNP	B	SE	*P*-value	OR (95% CI)
1	IL-2RA	10	–0.2352	0.0895	0.0086	0.790 (0.663–0.942)
2	Cystatin D	10	–0.0769	0.0345	0.0258	0.926 (0.865–0.991)
2	Artemin	10	0.083	0.039	0.034	1.086 (1.006–1.173)

CI = confidence interval, IL-2RA = interleukin-2 receptor alpha, NSNP = number of single nucleotide polymorphisms, OR= odds ratio, SE = standard deviation.

**Figure 6.**
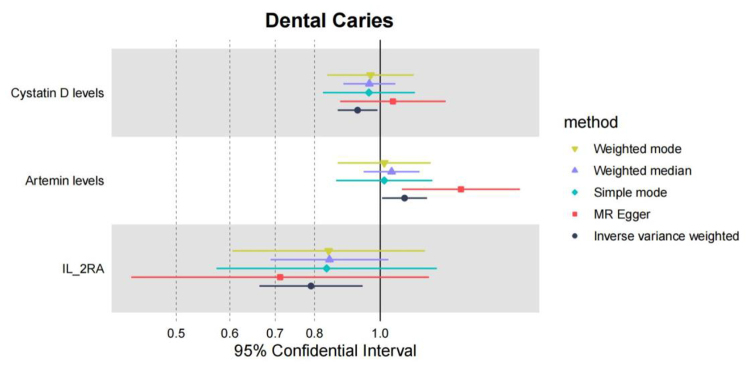
Forest plot of MR analysis. Forest plot to visualize the causal effect of oral cancer on the risk of cytokines by inverse variance weighted, MR-Egger regression, weighted median, weighted mode method, and simple mode. Dental caries may increase the risk of Artemin levels and decrease Cystatin D and IL-2RA levels. MR-Egger = MR-Egger regression.

The forest plot from 5 different MR methods is shown in Figure 6. Sensitivity analyses did not reveal significantly detrimental results for these findings (Table 4). The directionality was evaluated using the Steiger test and is shown in Table S8, Supplemental Digital Content 11. Bubble plots of reverse analysis MR analysis are shown in Figure 4C, D. Leave-one-out analysis is shown in Figure S4, Supplemental Digital Content 12. The funnel plots are shown in Figure S5, Supplemental Digital Content 13. Scatter plots for the identified cytokines across various tests are displayed in Figure S6, Supplemental Digital Content 14.

**Table 4:** Sensitivity analysis of dental caries on cytokines.

Cohort	Exposure	IVW Cochran	MR-PRESSO	Egger intercept
1	IL-2RA	0.108	0.143	0.674
2	Cystatin D	0.485	0.533	0.195
2	Artemin	0.602	0.657	0.077

IL-2RA: Interleukin-2 receptor alpha, IVW = inverse-variance weighted, MR-PRESSO = Pleiotropy RESidual Sum and Outlier.

## 4. Discussion

This study employed a bidirectional, 2-sample MR framework to dissect the causal interplay between genetically predicted levels of circulating cytokines and the risk of dental caries. By leveraging genetic variants as IVs, this approach circumvents the inherent limitations of confounding and reverse causation that plague traditional observational studies,^[7]^ thereby permitting more robust causal inference.^[27,28]^ Our analysis provides novel genetic evidence clarifying the complex and largely asymmetric relationship between systemic inflammatory mediators and the pathogenesis of dental caries. The principal findings indicate that while dental caries causally influence the levels of several systemic biomarkers, the reverse pathway is more selective, with only specific cytokine axes demonstrating a causal effect on caries risk.

Our forward MR analysis identified several cytokines with a causal influence on caries risk, implicating specific immune pathways in disease susceptibility. A genetically predicted increase in CXCL9 was associated with a higher risk of caries (OR = 1.251, 95% CI: 1.051–1.489). CXCL9 is a potent chemoattractant for T cells, natural killer cells, and other immune cells expressing the CXCR3 receptor.^[29]^ In the context of pulpitis, the inflammatory condition resulting from advancing caries, local tissue is characterized by the infiltration of CXCR3-positive T cells,^[30,31]^ and pulp fibroblasts can be induced to produce CXCL9’s sister chemokine, CXCL10.^[30]^ Our finding suggests that a lifelong, genetically determined elevation in systemic CXCL9 may “prime” the host for a more aggressive inflammatory response to cariogenic bacteria, accelerating the recruitment of effector lymphocytes into the dental pulp and exacerbating tissue damage and accelerating lesion progression.

Similarly, we identified causal risk effects for CD244 (OR = 1.196, 95% CI: 1.026–1.394), CTACK (OR = 1.150, 95% CI: 1.039–1.272), and CCL25 (OR = 1.136, 95% CI: 1.010–1.277). CD244 is a complex immunoregulatory receptor on NK and T cells whose signaling can be either activating or inhibitory, depending on the cellular context and availability of adaptor proteins.^[21,32]^ In chronic inflammatory settings, sustained CD244 signaling often becomes inhibitory, contributing to immune cell exhaustion.^[21]^ This could paradoxically promote caries progression by impairing an effective, controlled immune response against the microbial biofilm. The chemokines CCL27 and CCL25, acting through their respective receptors CCR10 and CCR9, are critical for T-cell trafficking to barrier tissues like the skin and gut mucosa.^[33,34]^ Expression of these receptors has been noted in inflamed pulp tissue, and our findings provide the first genetic evidence that these specific axes may be causally involved in orchestrating the immune infiltrate that drives caries-related pathology.

Conversely, our analysis revealed protective effects for RANTES (OR = 0.853, 95% CI: 0.761–0.957), IL-17 (OR = 0.830, 95% CI: 0.714–0.965), and IFN-γ (OR = 0.787, 95% CI: 0.645–0.961). The protective role of RANTES is noteworthy; while it is a pro-inflammatory chemokine, it is crucial for coordinating balanced T-cell responses, and its absence can exacerbate chronic infections.^[35]^ This finding aligns with observational data linking lower RANTES levels to developmental enamel defects, a known risk factor for caries.^[36]^ The protective effect of IL-17 is also intriguing, given its well-documented pro-inflammatory role in many diseases.^[37,38]^ However, the primary function of IL-17 is in mucosal host defense against bacteria and fungi, largely through the recruitment of neutrophils.^[39,40]^ A genetically determined capacity for a robust but well-regulated IL-17 response may confer protection by enabling efficient clearance of cariogenic bacteria at the earliest stages of colonization, preventing the establishment of a chronic lesion.^[39,40]^ This finding highlights the context-dependent role of IL-17; while potentially detrimental in the context of established, chronic inflammation (e.g., in periodontitis), its primary function in host defense may be paramount in preventing the initial establishment of a cariogenic biofilm. Finally, the protective effect of IFN-γ, a canonical Th1 cytokine, may stem from its role in activating macrophages for potent antimicrobial activity and in modulating T-cell homeostasis to prevent excessive, tissue-damaging inflammation within the pulp.^[41]^

The reverse MR analysis shows that genetic liability to dental caries causally alters the systemic immune profile. Specifically, caries was found to increase circulating levels of Artemin (OR = 1.086, 95% CI: 1.006–1.173). Artemin is a neurotrophic factor involved in neuronal survival and pain signaling, particularly cold pain, via its receptor GFRα3.^[42]^ Its upregulation has been documented in other chronic inflammatory and painful conditions, such as pancreatitis.^[43]^ Our finding suggests that the chronic neuro-inflammatory state of pulpitis, a direct consequence of caries, drives a systemic increase in this factor, potentially linking a local oral disease to systemic changes in pain-related pathways.

Furthermore, caries was found to causally decrease levels of Cystatin D (OR = 0.926, 95% CI: 0.865–0.991) and the IL-2RA (OR = 0.790, 95% CI: 0.663–0.942). Salivary cystatins are generally considered protective against caries, contributing to the enamel pellicle and exhibiting antimicrobial properties.^[44]^ A systemic reduction in Cystatin D in individuals genetically prone to caries may reflect a broader dysregulation of this protective protein family in response to the chronic disease process. The finding for IL-2RA is particularly significant. As the high-affinity component of the IL-2 receptor, IL-2RA is constitutively expressed on and is essential for the development, homeostasis, and suppressive function of regulatory T cells (Tregs).^[45]^ Our result suggests that the chronic inflammatory burden imposed by dental caries may causally lead to a systemic reduction in IL-2RA levels, which could impair Treg function and thereby contribute to the state of systemic, low-grade inflammation often observed in individuals with a high burden of chronic disease, a key tenet of the oral-systemic link.

Our findings both align with and extend the existing literature on the immunology of dental caries. Previous studies have primarily used histological or transcriptomic analyses to identify immune mediators like CXCL9 and IL-17 within carious lesions.^[30,31,46]^ While these studies established an association, our MR analysis provides the first genetic evidence for a causal role, shifting the paradigm from correlation to causation. Specifically, our identification of a protective role for IL-17 offers a nuanced perspective. While often implicated in inflammatory tissue damage in diseases like periodontitis,^[47]^ our results suggest that its function in neutrophil-mediated bacterial clearance at the enamel surface is causallyprotective against lesion initiation. This may help resolve conflicting reports on the role of Th17 responses in oral health and underscores the importance of investigating disease-specific immune functions.

Furthermore, the reverse MR findings provide a potential mechanistic underpinning for the well-documented oral-systemic link.^[48]^ Observational studies have consistently linked poor oral health to systemic inflammatory conditions, but the causal direction has been ambiguous. Our discovery that genetic liability to caries causally reduces systemic IL-2RA levels – a key molecule for regulatory T cell function – offers a novel pathway through which a chronic oral infection can promote systemic immune dysregulation. This supports the hypothesis that the oral cavity can act as a persistent source of inflammatory stimuli with systemic consequences, a concept increasingly recognized in recent literature.^[49]^ By establishing a causal direction from a specific oral disease to a fundamental systemic immune pathway, our study moves beyond association and provides a genetic basis for this important clinical connection.

The primary strength of this study is its bidirectional MR design, which provides a more comprehensive and robust assessment of causality than is possible with observational studies. However, several limitations must be acknowledged. First, the GWAS summary statistics used were derived predominantly from individuals of European ancestry, which may limit the generalizability of our findings to other populations. Replication in more diverse cohorts is a critical next step. Second, while we employed multiple sensitivity analyses to test for horizontal pleiotropy, its potential influence cannot be entirely excluded. Third, MR estimates reflect the effect of a lifelong genetic predisposition to altered cytokine levels, which may differ from the effects of acute, postnatal changes or therapeutic interventions.

## 5. Conclusion

In summary, this genetic study demonstrates that dental caries is a causal driver of systemic inflammation. It also reveals a reverse pathway where specific immune axes, such as CXCL9 and IL-17, contribute to caries etiology. This reinforces the oral-systemic connection and calls for future research to functionally validate these pathways, which could pave the way for new cytokine-based therapies and biomarkers for dental caries.

## Acknowledgments

We express our gratitude to all SCALLOP Consortium and FinnGen studies, QTLbase working group, GWAS Catalog for providing open access to the summary association statistics data.

## Author contributions

**Conceptualization:** Ziyang Hu, Ping Shi.

**Data curation:** Fengzhen Lei.

**Formal analysis:** Fengzhen Lei.

**Investigation:** Zhe Xu, Xiaofen Liu.

**Methodology:** Zhe Xu, Ziyang Hu, Ping Shi.

**Project administration:** Ziyang Hu, Ping Shi.

**Resources:** Zhe Xu, Xiaofen Liu, Fengzhen Lei, Ziyang Hu.

**Software:** Zhe Xu, Xiaofen Liu, Ziyang Hu.

**Supervision:** Ziyang Hu.

**Visualization:** Zhe Xu, Ping Shi.

**Writing – original draft:** Zhe Xu.

**Writing – review & editing:** Ziyang Hu, Ping Shi.




























