# Supplementary figures and images for: Genetic evidence for causal link between systemic inflammation and dental caries risk: A bidirectional Mendelian randomization study
Source: Medicine (Baltimore). 2026 Jul 31;105(31):e50025. doi: 10.1097/MD.0000000000050025 (PMC13433123; doi:10.1097/MD.0000000000050025)

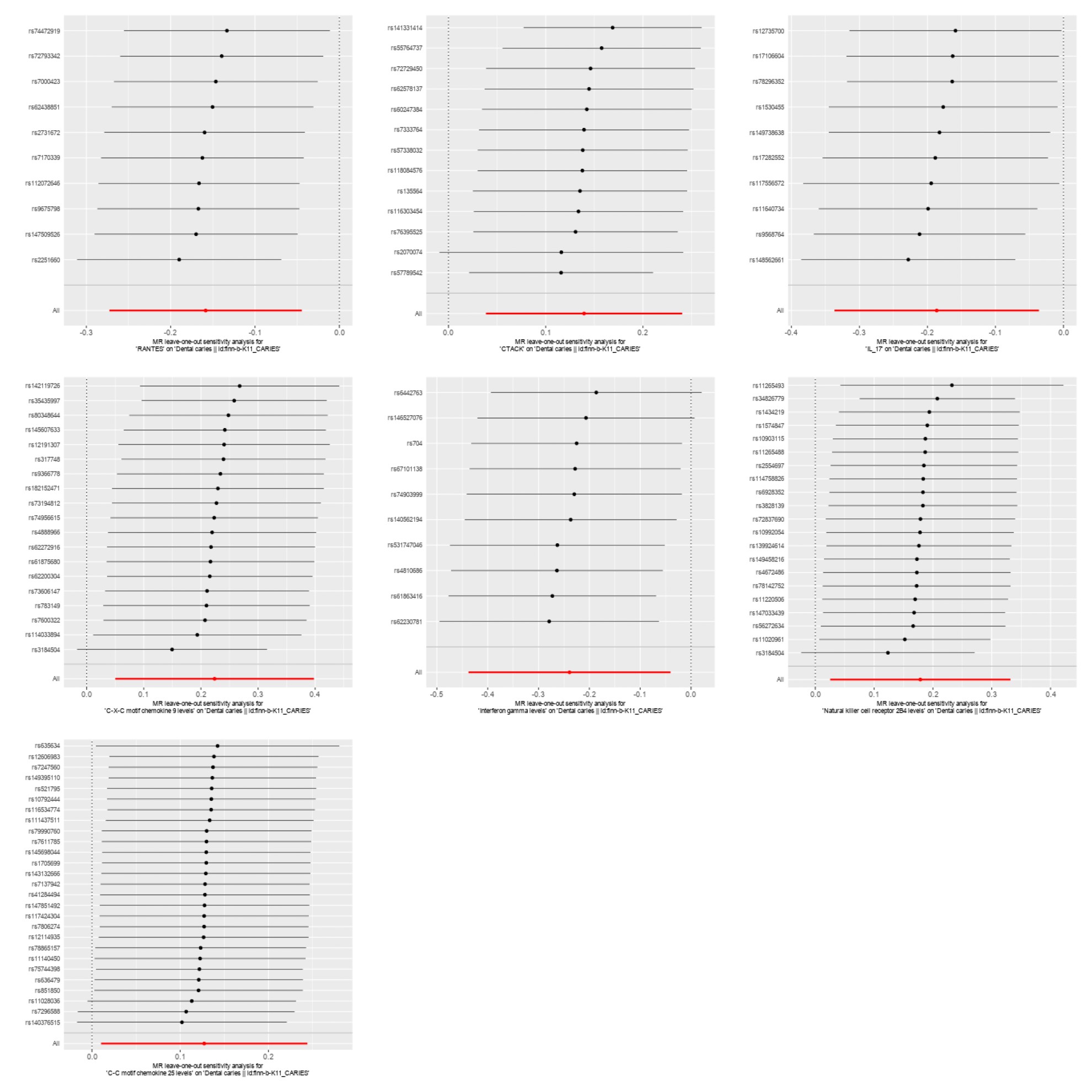

Supplement: Supplementary file 6 [file medi-105-e50025-s006.jpg]

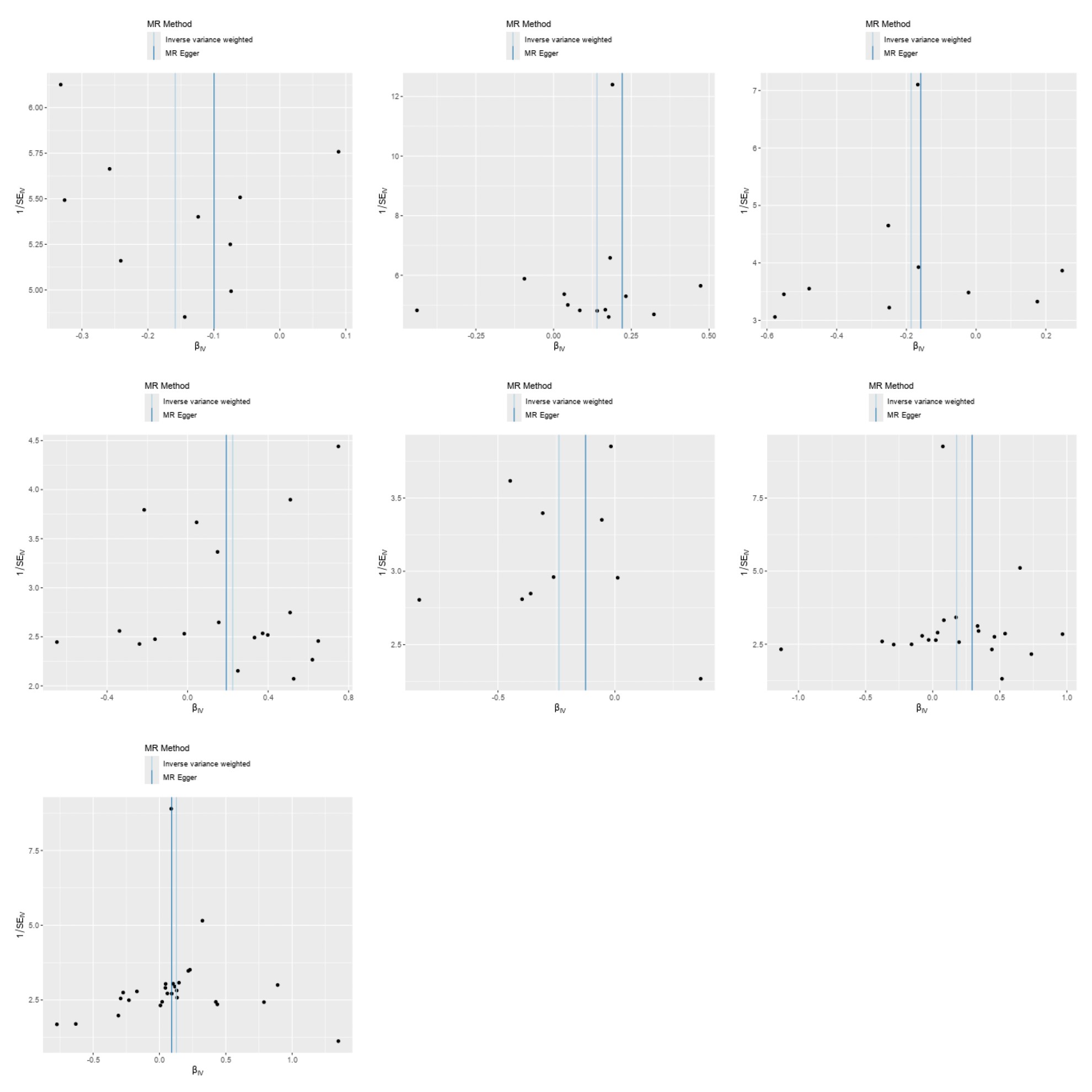

Supplement: Supplementary file 7 [file medi-105-e50025-s007.jpg]

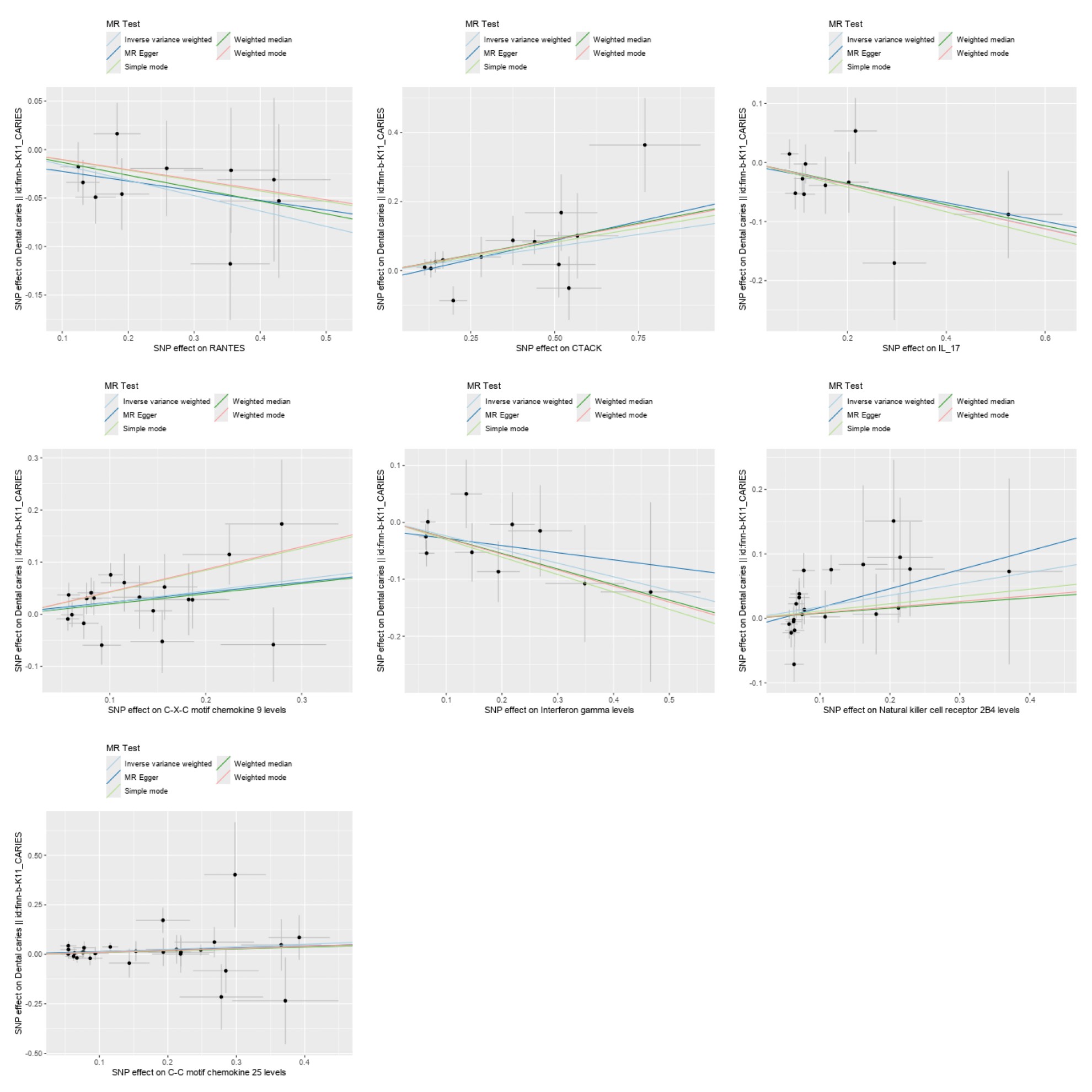

Supplement: Supplementary file 8 [file medi-105-e50025-s008.jpg]

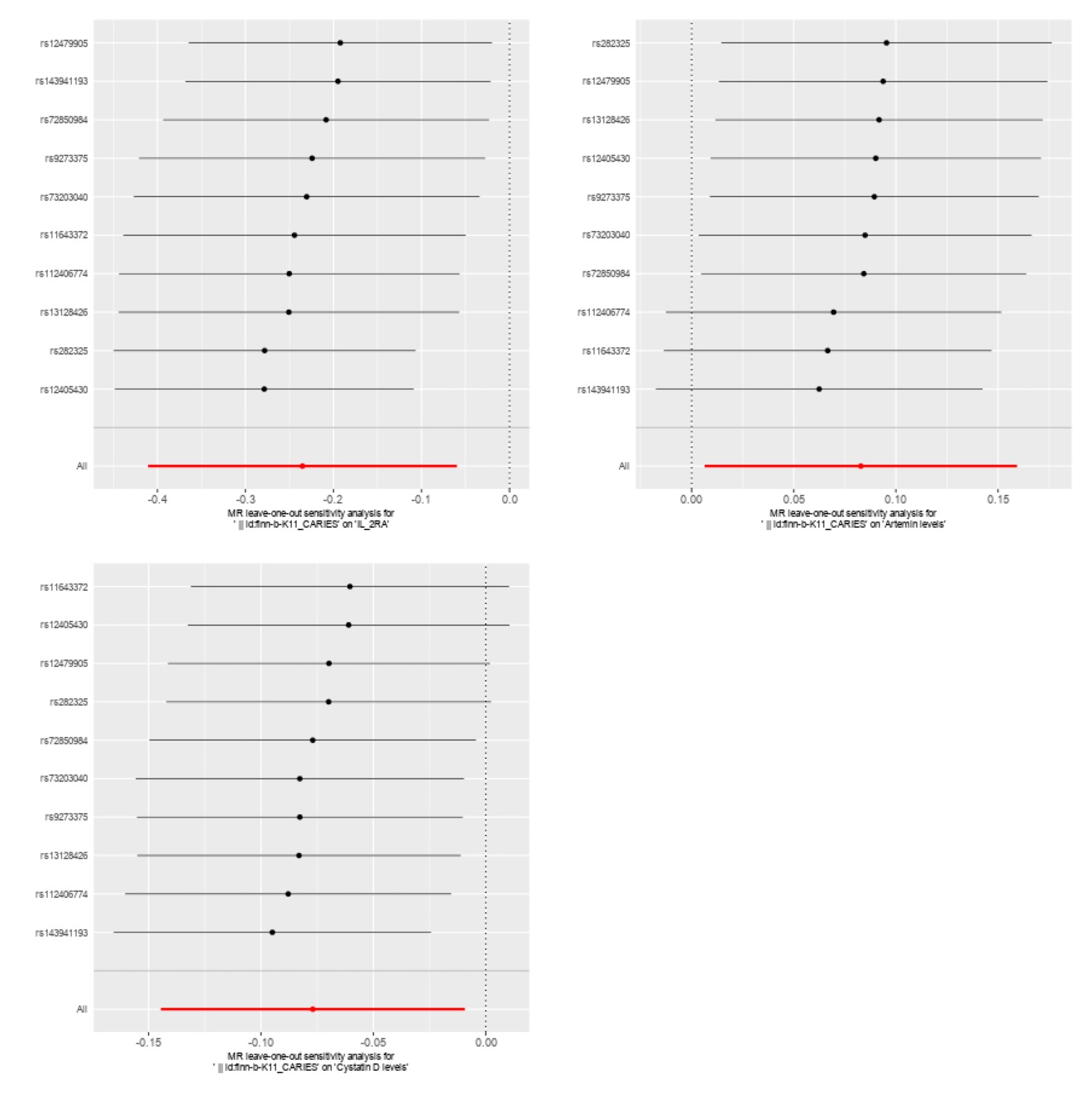

Supplement: Supplementary file 12 [file medi-105-e50025-s012.jpg]

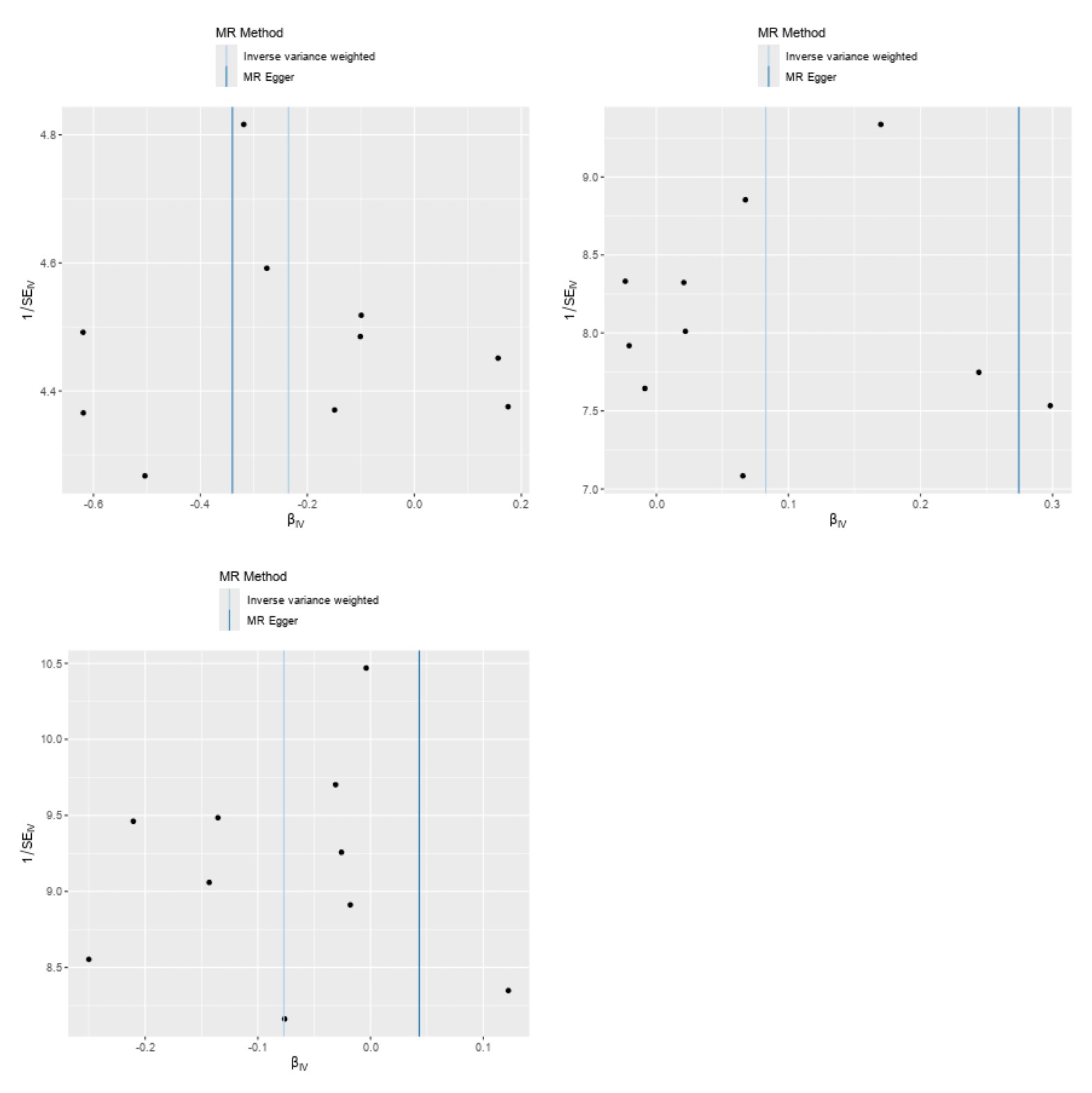

Supplement: Supplementary file 13 [file medi-105-e50025-s013.jpg]

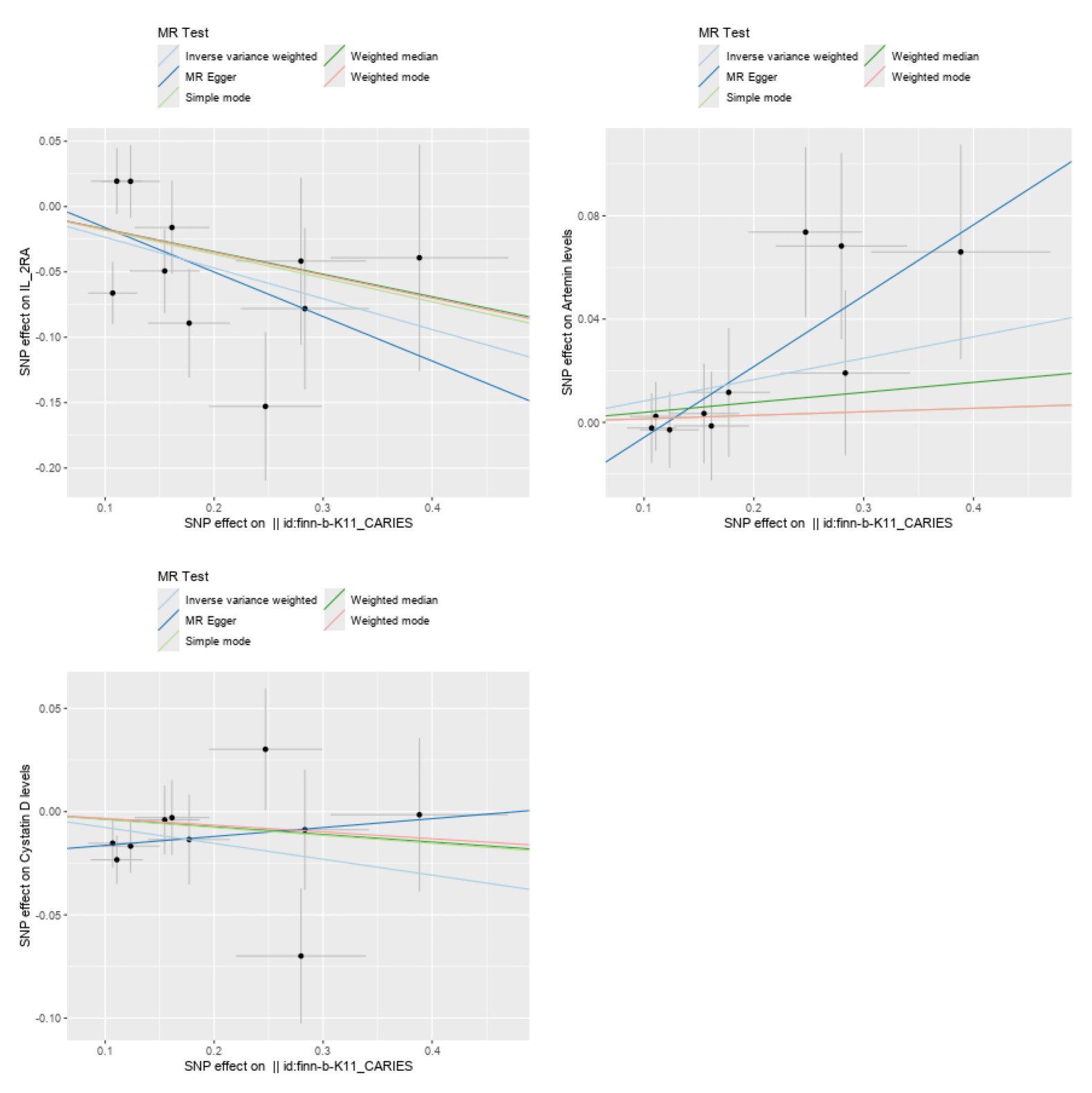

Supplement: Supplementary file 14 [file medi-105-e50025-s014.jpg]
